# Immunostaining of modified histones defines high-level features of the human metaphase epigenome

**DOI:** 10.1186/gb-2010-11-11-r110

**Published:** 2010-11-15

**Authors:** Edith Terrenoire, Fiona McRonald, John A Halsall, Paula Page, Robert S Illingworth, A Malcolm R Taylor, Val Davison, Laura P O'Neill, Bryan M Turner

**Affiliations:** 1 Chromatin and Gene Expression Group, Institute of Biomedical Research, College of Medical and Dental Sciences, University of Birmingham, Edgbaston, Birmingham B15 2TT, UK; 2 West Midlands Regional Genetics Laboratory, Birmingham Women's NHS Foundation Trust, Metchley Park Road, Edgbaston, Birmingham B15 2TG, UK; 3 Current address: School of Biosciences, University of Birmingham, Edgbaston, Birmingham B15 2TT, UK; 4 The Wellcome Trust Centre for Cell Biology, University of Edinburgh, The King's Buildings, Edinburgh EH9 3JR, UK; 5 Current address: MRC Human Genetics Unit, Crewe Road, Edinburgh EH4 2XU, UK; 6 School of Cancer Sciences, College of Medical and Dental Sciences, University of Birmingham, Edgbaston, Birmingham B15 2TT, UK

## Abstract

**Background:**

Immunolabeling of metaphase chromosome spreads can map components of the human epigenome at the single cell level. Previously, there has been no systematic attempt to explore the potential of this approach for epigenomic mapping and thereby to complement approaches based on chromatin immunoprecipitation (ChIP) and sequencing technologies.

**Results:**

By immunostaining and immunofluorescence microscopy, we have defined the distribution of selected histone modifications across metaphase chromosomes from normal human lymphoblastoid cells and constructed immunostained karyotypes. Histone modifications H3K9ac, H3K27ac and H3K4me3 are all located in the same set of sharply defined immunofluorescent bands, corresponding to 10- to 50-Mb genomic segments. Primary fibroblasts gave broadly the same banding pattern. Bands co-localize with regions relatively rich in genes and CpG islands. Staining intensity usually correlates with gene/CpG island content, but occasional exceptions suggest that other factors, such as transcription or SINE density, also contribute. H3K27me3, a mark associated with gene silencing, defines a set of bands that only occasionally overlap with gene-rich regions. Comparison of metaphase bands with histone modification levels across the interphase genome (ENCODE, ChIP-seq) shows a close correspondence for H3K4me3 and H3K27ac, but major differences for H3K27me3.

**Conclusions:**

At metaphase the human genome is packaged as chromatin in which combinations of histone modifications distinguish distinct regions along the euchromatic chromosome arms. These regions reflect the high-level interphase distributions of some histone modifications, and may be involved in heritability of epigenetic states, but we also find evidence for extensive remodeling of the epigenome at mitosis.

## Background

Large scale projects are underway to map the epigenomes of various eukaryotes, including humans. The objective is usually to define the distribution across the genome of modified histones, various non-histone proteins or methylated cytosines, and then link these modifications to genomic functions [1-3]. Genome-wide analyses have been made possible by coupling the long-established technique of chromatin immunoprecipitation (ChIP) with either high density DNA microarrays (ChIP-chip) or next-generation DNA sequencing (ChIP-seq) [4]. These powerful technologies require material from large numbers of cells and the data generated inevitably represent a mean value derived from cells with differing patterns of expression from a significant subset of genes. Differences can arise through intrinsic transcriptional noise or because cells are in different phases of the cell cycle. Such cell to cell heterogeneity inevitably limits the precision with which histone modifications can be linked to chromatin function. In principle, this issue can be addressed by using immunomicroscopy to examine the distribution of histone modifications at the single cell level. Metaphase chromosome spreads provide a source of material in which individual chromosomes can be identified and in which the entire human epigenome can be scanned in a single cell. This approach has several additional advantages: there is little or no transcription at metaphase, removing a major source of variability between cells, consistency from cell to cell can be monitored, fluorescent probes are extremely sensitive (offering detection at the single gene level if required) and the procedure is quick (once experimental conditions are established) and relatively cheap. It should also be noted that immunostaining, if properly controlled, can detect modified histones and other proteins across the entire genome, including repeat-rich regions that are inaccessible to sequencing-based approaches [4]. While microscopy cannot match the ultimate resolving power of ChIP-seq, it has the potential to provide a valuable complementary approach to epigenomic mapping.

Immunolabeling of metaphase chromosomes is a well established technique and has revealed dramatic regional differences in the distribution of specific histone modifications, particularly the distinctive pattern of modifications present on centric (constitutive) heterochromatin in plants and animals [5-7] and the facultative heterochromatin of the inactive X chromosome in female mammals [8,9]. Immunolabeling of meiotic (pachytene) chromosomes in maize has shown regional variation in levels of various methylated histone isoforms, with distinctive differences between heterochromatin and euchromatin [10].

Surprisingly, there has been only limited use of metaphase chromosome immunostaining to map histone modifications across individual chromosomes [11,12], and no systematic attempt to explore the genome-wide distribution of multiple histone modifications.

Here we describe a systematic analysis of the distribution of selected histone modifications across metaphase chromosomes from normal human cells. Antibodies to histone modifications previously linked to active transcription (H3K9ac, H3K27ac and H3K4me3, described collectively as active modifications) all highlight the same 10- to 50-Mb genomic regions, giving a characteristic and consistent banding pattern. Bands closely correspond to regions rich in genes and CpG islands (CGIs). In contrast, H3K27me3, a mark associated with gene silencing, shows a preference for telomeric regions and defines bands that only occasionally overlap with gene rich regions. At 10-Mb resolution, active modifications have similar, though not identical, distributions across interphase [13] and metaphase chromosomes, while H3K27me3 is clearly different. The results suggest that there is extensive remodeling of the epigenome as cells enter mitosis, but that a high-level memory of some components of the interphase epigenome is retained into metaphase.

## Results

### Classification of unfixed metaphase chromosomes

Standard protocols for preparation and staining of metaphase chromosomes require fixation in acidified organic solvents, a step that extracts the great majority of histones and other proteins [14]. We have adopted an approach using unfixed chromosomes [9,15,16], a procedure that has the major advantage that histones remain in their native (that is, unfixed, undenatured) form and are therefore structurally compatible with the synthetic peptides used to raise anti-histone antisera [17,18]. We found that both the relative sizes and centromeric indices (arm ratios) of unfixed chromosomes were very similar to their counterparts fixed in methanol/acetic acid (Additional files 1 and 2), allowing us to use these properties as a first step in chromosome identification. Unfixed chromosomes are not amenable to conventional G-banding procedures. To distinguish morphologically similar chromosomes, we used the chromosome-specific banding patterns generated by the DNA counterstain DAPI (4,6-diamino-2-phenyl-indole). DAPI selectively stains regions that are AT-rich and GC-poor, and gives a banding pattern that resembles G-banding and is unique for each chromosome [17].

### Modifications associated with transcriptionally active and silent chromatin show distinctive, banded distributions across metaphase chromosomes

**Figure 1:**
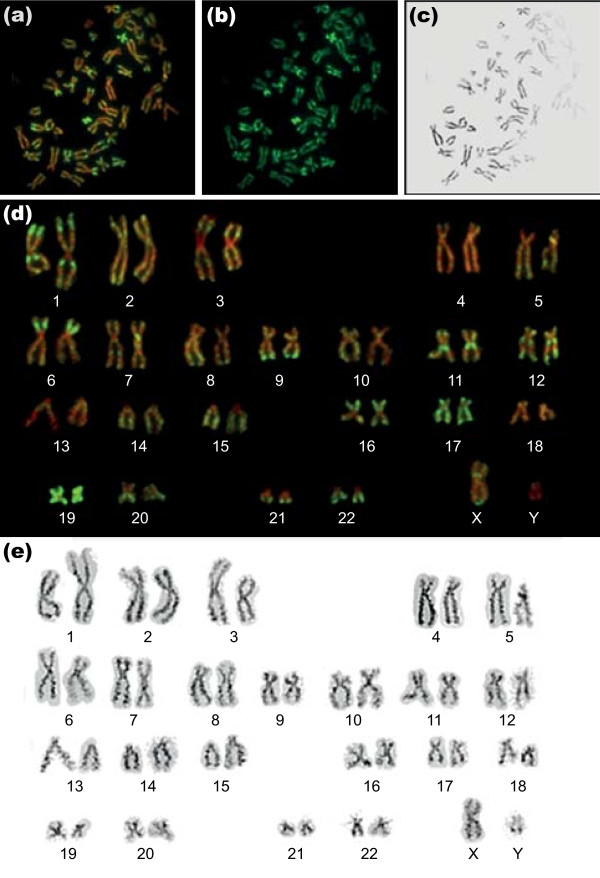
**Distribution of H3K4me3 across human metaphase chromosomes**. **(a-c) **Metaphase chromosome spreads from human lymphoblastoid cells immunostained with antibodies to H3K4me3 (fluorescein isothiocyanate (FITC), green) and counterstained with DAPI (pseudocolored red). Panel (a) shows both stains, panel (b) FITC only and panel (c) DAPI only, shown in black to resemble conventional G-banding. **(d) **Immunostained karyotype constructed from the chromosome spread shown in (a-c). **(e) **Reverse DAPI (rDAPI) karyotype constructed from the same spread.

Unfixed metaphase chromosome spreads from human lymphoblastoid cells were immunostained with antibodies to histone H3 tri-methylated at lysine 4 (H3K4me3), a modification that has been associated with transcriptionally active, or potentially active, chromatin [18-21]. Centromeric heterochromatin was consistently unstained, while the arms of most chromosomes showed a characteristic pattern of brightly stained and weakly stained regions (Figure 1a, b). Using a combination of size, centromeric index and reverse DAPI banding (Figure 1c), we were able to identify all chromosomes and construct karyotypes (Figure 1d, e). There was consistently strong staining of both arms of chromosome 19, weak staining of chromosome 13 and distinctive banding of most chromosomes, with particularly prominent bands on chromosomes 1, 6, 9, 11 and 12. The immunofluorescent banding pattern was consistent between sister chromatids and homologues and reproducible from one spread to another, despite the inevitable differences in overall chromosome size. Alignments of chromosomes from five immunostained spreads are shown in Additional file 3.

**Figure 2:**
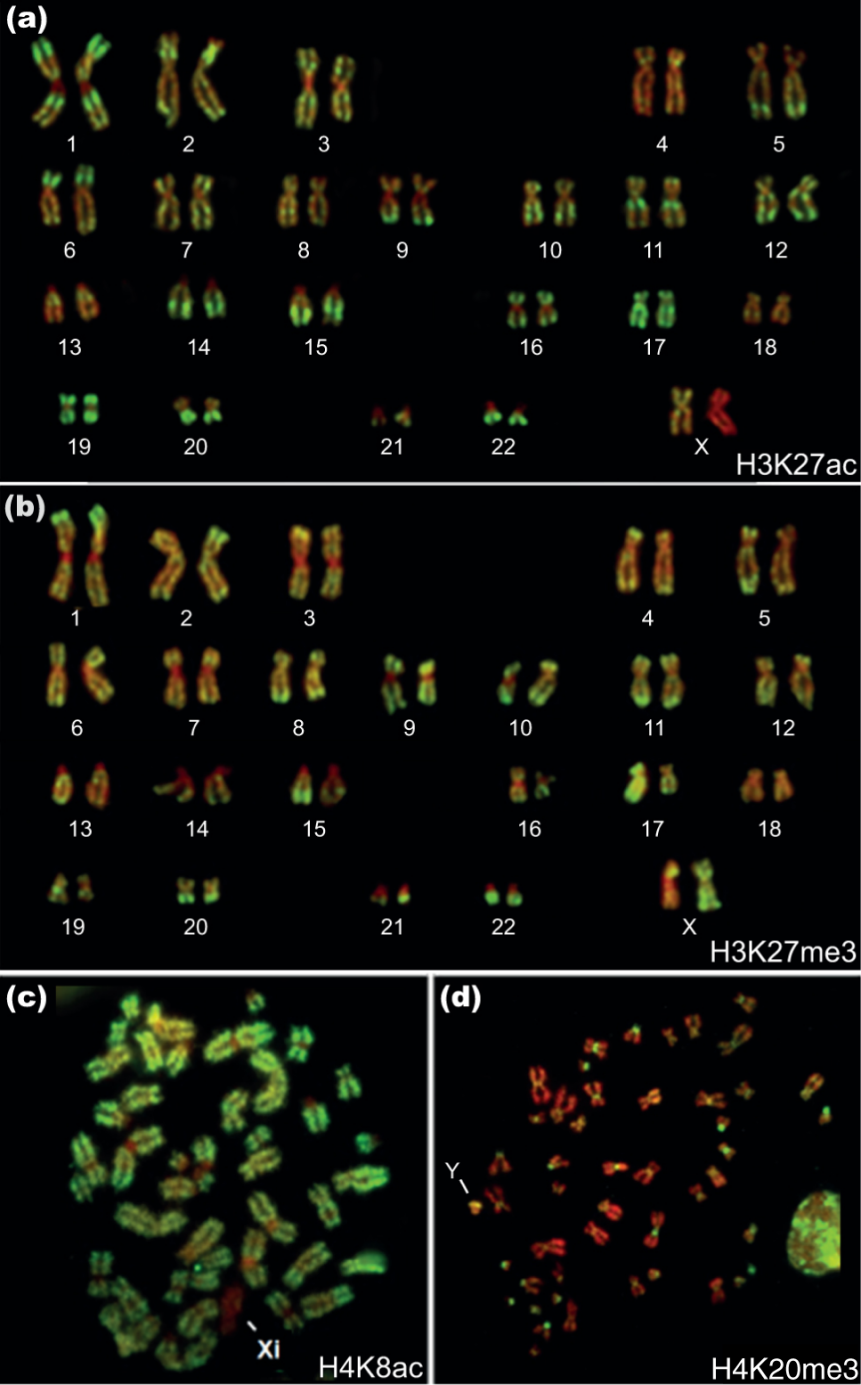
**Immunolabeling of metaphase chromosomes from human lymphoblastoid cells with antisera to H3K27ac, H3K27me3, H4K8ac and H4K20me3**. **(a) **Immunostained karyotype from a metaphase chromosome spread immunostained with antibodies to H3K27ac (fluorescein isothiocyanate (FITC), green) and counterstained with DAPI (pseudocolored red). **(b) **Immunostained karyotype from a metaphase chromosome spread immunostained with antibodies to H3K27me3 (FITC, green) and counterstained with DAPI (pseudocolored red). **(c) **Metaphase chromosome spread immunostained with antibodies to H4K8ac (FITC, green) and counterstained with DAPI (pseudocolored red). Note the complete absence of FITC labeling at centric (constitutive) heterochromatin and the facultative heterochromatin of the inactive X (Xi). **(d) **Metaphase chromosome spread immunostained with antibodies to H4K20me3 (FITC, green) and counterstained with DAPI (pseudocolored red). Note the extensive, patchy staining of the interphase nucleus on the right. The arms of the Y chromosome (indicated) are labeled but its centric heterochromatin is not.

Very similar immunostaining patterns were given by antisera to two other modifications also loosely associated with transcriptionally active chromatin, namely H3 acetylated at lysine 27 (H3K27ac) and H3 acetylated at lysine 9 (H3K9ac) [22,23] (Figure 2a; Additional files 4 and 5). Conversely, staining with a variety of antisera to acetylated H4 was more uniform. The acetylated H4 bands corresponded to those seen with antisera to H3K4me3 but the differential labeling of bands and interband regions was less extreme. A typical example is shown in Figure 2c. H4K8ac is absent from both constitutive (centric) and facultative heterochromatin and our findings are generally consistent with previous studies on acetylated H4 [10,13].

H3 tri-methylated at lysine 27 (H3K27me3) is put in place by the methyltransferase Ezh2, a component of the Polycomb silencing complex PRC2 and has been associated with formation of facultative heterochromatin and gene silencing [24-26]. In female cells, one of the two X chromosomes generally stained more strongly than its homologue, and more strongly than the single X in male cells (Figure 2b; Additional file 6). The more intensely stained X is likely to be the inactive homologue [27]. H3K27me3 was undetectable on blocks of constitutive centric heterochromatin (Figure 2b; Additional file 6) or on the Y heterochromatin in male cells (Additional file 7). There are distinctive regional variations in H3K27me3 staining intensity along the chromosome arms, but without the sharply defined banded distribution typical of H3K4me3 (Figure 1). We find only limited overlap between the two modifications. For example, the short arm of chromosome 6 is relatively enriched in both modifications, but on closer inspection H3K27me3 has a more telomeric location (6pter-22.3) than H3K4me3, which is centrally located in the short arm (centered at 6p21), leaving the telomeric region relatively weakly stained (compare the multiple examples of chromosome 6 in Additional files 3 and 6). Also, the prominent H3K4me3 band on chromosome 11q just below the centromere (11q12.1-13.3) is not enriched in H3K27me3 (Figure 2b). Overall, we find that H3K27me3 is consistently enriched at telomeric regions, at least on the larger chromosomes (chromosomes 1 to 15). This distinctive staining pattern was seen with two different antisera to H3K27me3 (listed in Additional file 8). H3K27ac is a modification that may act as an antagonist of Polycomb-mediated silencing through suppression of H3K27 tri-methylation [4,24]. While the distribution of H3K27ac (Figure 2a) is clearly different from that of H3K27me3 (Figure 2b), H3K27me3 is not consistently excluded from regions rich in H3K27ac, or *vice versa*.

Immunostaining with antibodies to H4 tri-methylated at lysine 20 (H4K20me3) strongly and selectively labeled the centric heterochromatin of metaphase chromosomes from human lymphoblastoid cells (Figure 2d), consistent with previous results in other cell types [6]. Absence of staining of centric heterochromatin by antisera to the other histone modifications tested here is clearly not due to a general inaccessibility of histone epitopes in heterochromatin. Chromosome arms were essentially unstained by antibodies to H4K20me3, with the exception of the Y chromosome in male cells, on which heterochromatic regions on the distal long arm were consistently stained (Figure 2d).

### Immunofluorescent chromosome banding in primary fibroblasts closely resembles that in lymphoblastoid cells

Over the course of the work presented here, complete immunostained karyotypes for H3K4me3, H3K9ac, H3K27ac and H3K27me3 have been constructed from lymphoblastoid cell lines (LCLs) derived from two different individuals, one male and one female. At the present level of resolution, we have found no evidence for individual differences in chromosome banding. The same banding patterns have also been seen in occasional preparations from two other LCLs (results not shown). Analyses of other cell types have been less extensive, but immunostaining of chromosomes from human primary fibroblasts with antibodies to H3K4me3 revealed a banding pattern essentially the same as that seen in LCLs (Additional file 9). The banding patterns described are not restricted to a particular cell lineage. However, differences may occur among more widely divergent, or aberrant, cell types. Improved resolution of immunofluorescent bands, perhaps through analysis of extended, prophase chromosomes, may also reveal differences not apparent with the present approach.

### Modifications associated with active chromatin are enriched in regions rich in genes and CpG islands

**Figure 3:**
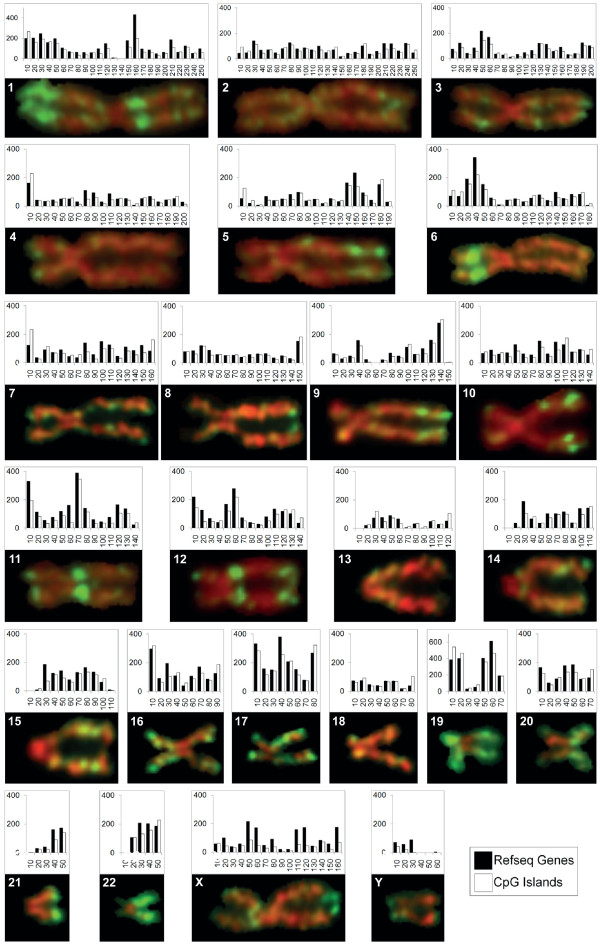
**Correspondence between gene density, CpG island density and H3K4me3 levels across human metaphase chromosomes**. Metaphase chromosomes from human lymphoblastoid cells immunostained with antibodies to H3K4me3 are aligned with histograms showing the distribution of genes (filled bars) and CpG islands (open bars) across the same chromosome. The example of each immunostained chromosome shown was selected, for clear and representative banding, from the chromosomes aligned in Additional file 3.

Recent analyses have confirmed that most genes are clustered in a relatively small number of genomic regions [28-30]. These gene-rich regions are also enriched in CGIs, relatively CpG-rich DNA sequences found at and around the promoter regions of many genes and characterized by low levels of DNA methylation [31,32]. We constructed gene density/CGI maps for each human chromosome by calculating the gene and CGI content of 10-Mb windows across the chromosome. In Figure 3, the resulting histograms are aligned with a representative example of each chromosome immunostained for H3K4me3. There is a close and consistent correspondence between high levels of H3K4me3 and regions of relatively high gene/CGI content. This is true not only for regions of intense staining (for example, landmark bands on chromosomes 1q, 6p and 11q) but also for less strongly staining bands that do not stand out in the original spreads (for example, the bands distributed across chromosomes 3 and 12) (Figure 1; Additional file 3). As expected from our earlier results, chromosomes immunostained with antibodies to H3K9ac and H3K27ac showed essentially the same close relationship between staining intensity and gene/CGI density (results not shown). In contrast, on chromosomes immunostained for H3K27me3, there was only limited overlap between gene/CGI-rich regions and staining intensity (Additional file 7).

**Figure 4:**
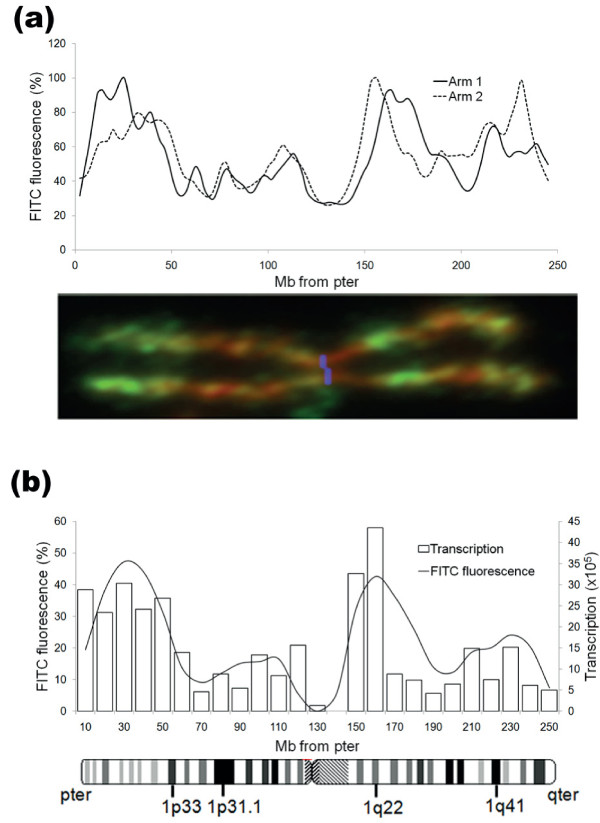
**Quantitative analysis of H3K4me3 across metaphase chromosome 1 and comparison with interphase transcription**. **(a) **Scanning of human chromosome 1 immunostained with antibodies to H3K4me3. Scans from each sister chromatid are shown (dotted and solid lines). The blue line on the immunostained chromosome was inserted manually to mark the centromere prior to scanning (see Materials and methods for details). Note that peak positions differ slightly between sister chromatids, presumably due to differential stretching during preparation. **(b) **Transcription from 3,071 RefSeq genes across chromosome 1 in human LCLs was measured by expression microarray and summed within 10-Mb windows across the chromosome. Transcription (open bars) is plotted as the sum of normalized gene expression values per 10-Mb window. H3K4me3 levels across chromosome 1 (solid line) were obtained by scanning (a). To obtain the mean distribution shown, each scanned chromatid was divided linearly into 25 equal segments (nominally 10 Mb each) and fluorescence values within each segment (expressed as percent maximum value for that scan) were summed. Each value shown is the average of 24 chromatids. The minimum value at the centromere (120 to 130 Mb) was used as a background value and subtracted. There is some broadening of peaks derived from multiple scans compared to single chromatid scans because of the shifts in peak position caused by differential stretching (a). A standard chromosome 1 ideogram showing major G bands is aligned with the histogram. FITC, fluorescein isothiocyanate.

To allow a quantitative analysis of the relationship between the distribution of histone modifications at metaphase and other chromosome properties, we measured the level of H3K4me3 across chromosome 1 by scanning. Typical scans of sister chromatids are shown in Figure 4a. Fluorescence intensity is expressed as a percentage of the most highly fluorescent element and distance along the chromosome is expressed in megabases (chromosome 1 is 247 Mb long and we have assumed a linear relationship between positions on the metaphase chromosome and genomic DNA). To allow us to combine data from multiple scans, the chromosome was divided into 25 equal segments (each having a nominal length of 10 Mb) and the total fluorescence within each segment calculated. The fluorescence distribution (banding pattern) obtained by averaging scans from 12 chromosomes (24 chromatids) is shown in Figure 4b. Comparison of these quantitative data with gene and CGI frequencies across chromosome 1, also grouped within 10-Mb windows (Figure 3), shows that they are closely correlated (r = 0.70 and 0.68 respectively, *P *< 0.0002).

As a first step in exploring the link between H3K4me3 levels at metaphase and transcription in interphase, we used single color, high-density oligonucleotide arrays to measure transcript levels for 3,071 RefSeq genes across chromosome 1 in the same lymphoblastoid cells used for immunolabeling. Total transcript levels within 10-Mb windows across chromosome 1 are shown in Figure 4b. There is a close correlation between interphase transcription and levels of H3K4me3 at metaphase, measured by immunofluorescence labeling (Figure 4b; r = 0.73, *P *< 0.00002). Both transcription and H3K4me3 immunofluorescence are strongest in regions of the chromosome depleted in major G bands (for example, 1pter-p33, 1q21-23; Figure 4a, b).

### Genome-wide distribution of histone modifications in interphase and metaphase cells

**Figure 5:**
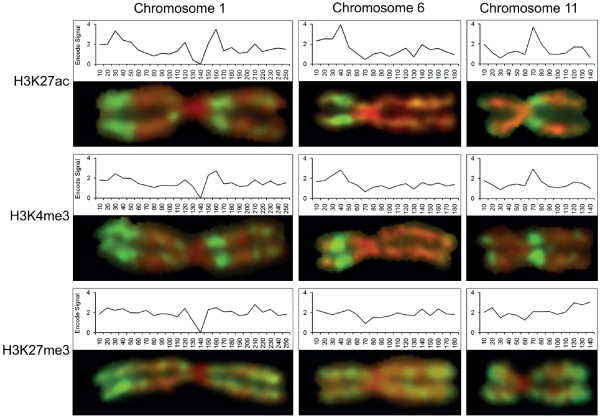
**Comparison of histone modifications across interphase and metaphase chromosomes**. Representative metaphase chromosomes immunostained for H3K27ac, H3K4me3 and H3K27me3 are aligned with the distribution of the same modification across the equivalent interphase chromosome assembled from ENCODE ChIP-seq data [13]. Graphs were constructed by adding the number of reads within 10-Mb windows, as used to plot gene/CGI frequencies (Figure 3), transcript levels and fluorescein isothiocyanate (FITC) staining intensity (Figure 4).

The genome-wide distribution of various histone modifications in asynchronous (mostly interphase) human lymphoblastoid cells has recently been defined by ChIP-seq [33] (see Materials and methods). The results can be aligned with immunostained metaphase chromosomes to provide an initial comparison of the interphase and metaphase epigenomes. Results for three modifications (H3K27ac, H3K4me3 and H3K27me3) on three chromosomes (chromosome 1, 6 and 11) are shown in Figure 5. At 10-Mb resolution, there is a close correspondence between the interphase and metaphase distributions of H3K27ac and H3K4me3, with clearly defined interphase peaks aligning with the major metaphase bands. The correspondence for H3K4me3 is particularly precise, with even the weakly stained double band on distal chromosome 1q evident in interphase (Figure 5). Quantitative analysis using chromosome scanning data (Figure 4) confirms the visual alignment of H3K4me3 levels across chromosome 1 at metaphase and interphase, with a strong correlation between them (r = 0.74, *P *< 0.00002; all pairwise correlations are presented in Additional file 10). In contrast, we find little correspondence between the distributions of H3K27me3 in interphase and metaphase. The chromosome-wide distribution of H3K27me3 in interphase at 10-Mb resolution is relatively homogeneous, the most prominent feature being its depletion across the block of centric heterochromatin on chromosome 1 (Figure 5). There are no interphase peaks corresponding to the highly stained H3K27me3 bands present at metaphase.

Previous studies have shown that progression into mitosis is accompanied by an overall decrease in global histone acetylation levels, reduced acetate turnover and changes in the relative levels of acetylation at specific lysines [34,35]. In view of this, it is perhaps surprising that the high level distribution of histone acetylation across the interphase genome, as revealed by ChIP-seq, is retained in metaphase chromosomes (Figure 5). A possible explanation comes from the finding that for both H3K27ac and H3K4me3, the differences between enriched and depleted regions are more extreme in metaphase chromosomes than in interphase chromatin. For example, the regions on chromosome 1p and 1q that lie between the brightly stained bands (distal 1p, proximal 1q) are virtually unstained and comparable to centric heterochromatin, a finding confirmed for H3K4me3 by quantitative scanning (Figure 4a). The equivalent regions at interphase show levels of modification well above that of centric heterochromatin (Figure 5). While the different technologies used to derive the two sets of data may contribute to these differences, the comparison suggests that at least some histone modifications are preferentially removed from gene-poor chromosomal regions as cells enter mitosis.

### Histone modification and genomic features

**Figure 6:**
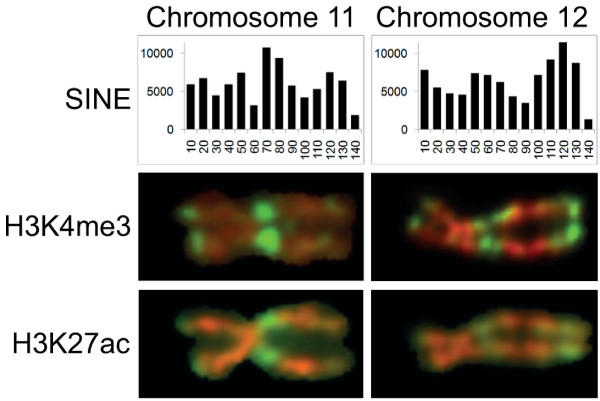
**Correspondence between SINE repeat frequency and levels of H3K4me3 and H3K27ac across human chromosomes 11 and 12**. Metaphase chromosomes 11 and 12 from human lymphoblastoid cells immunostained with antibodies to H3K4me3 or H3K27ac, as indicated, are aligned with histograms showing the distribution of SINE repeat sequences across the same chromosome. The examples of immunostained chromosomes shown were selected, for clear and representative banding, from the chromosomes aligned in Additional files 3 and 4. Repeat masker-defined SINE repeats were taken from USCS (hg18) human genome build [13] and allocated to 10-Mb windows spanning each chromosome.

H3 di-methylated at lysine 4 (H3K4me2) has been shown to be strongly enriched at promoters with the highest CpG content (CGI promoters), even when they are transcriptionally silent [36]. It has been suggested that H3K4 methylation protects these promoters from silencing by CpG methylation, a proposition supported by *in vitro *experiments [37]. In light of these findings, one could propose that H3K4me3 levels at metaphase are a simple reflection of CGI density. However, closer inspection of the chromosome labeling patterns suggests that banding is unlikely to be solely attributable to simple genomic features such as gene or CGI density. For example, the gene-rich, CGI-rich region 11q12.1-13.3 is consistently one of the most strongly stained regions in the genome with antisera to the three activating modifications tested. The region at 11pter-15.3 is similarly gene-rich and only slightly less CGI-rich (Figure 3), yet stains much less strongly with antisera to H3K27ac and H3K4me3 (Additional files 3, 4 and 5). Another example is provided by the gene/CGI-rich regions across chromosome 12. The region on the q arm adjacent to the centromere labels with antisera to all three active modifications tested, but the labeling intensity is consistently less than, or at best equal to, labeling of the less gene/CGI-rich region on the distal q arm (Figure 3; Additional files 3, 4 and 5). It is interesting to note that this strongly staining distal region has a higher density of short interspersed nuclear element (SINE) repeats (UCSC hg18 [13]) than the more gene-rich, centromere proximal region (Figure 6). This is unusual because gene/CGI density and SINE density are very closely correlated across the genome (figures for chromosome 1 are shown in Additional file 10). On the basis of these examples, it could be argued that SINE density is more closely associated with levels of active histone modifications than gene/CGI density. This possibility is supported by the correlations derived from the chromosome 1 scanning data (Additional file 10).

## Discussion

### Levels of genome organization

Immunostaining of polytene chromosomes from the salivary glands of chironimid insects established the principle that levels of histone acetylation across the interphase genomes of higher eukaryotes show extreme regional variation, giving distinctive and reproducible immunofluorescent banding patterns [38,39]. Islands of acetylated histone H4 occurred within transcriptionally active and silent regions and within condensed (phase dense) and more open (phase light) chromatin, and were therefore not solely dependent on either transcription or chromatin compaction [39]. In the absence of polytene chromosomes, it is only comparatively recently that the same principle has been shown to apply to the interphase genomes of mammals. By combining ChIP with a cloning strategy based on the serial analysis of gene expression (SAGE) technique, Roh *et al*. [40] identified over 46,000 regions enriched in H3 acetylated at lysine 9 and/or 14 in human T-cells. These regions, designated 'acetylation islands', were often associated with promoters, putative control elements and CGIs. At least some of the acetylated islands were dynamic features; activation of T cells with accompanying gene activation and chromatin remodeling resulted in the appearance of over 4,000 new islands and the disappearance of some pre-existing ones [40]. There was a close correlation between the frequency of acetylated islands and gene density [40] and in a chromosome-by-chromosome presentation of the data (supplementary Figure 3 in [40]), regions of high acetylation (for example, on chromosomes 1q, 6p, 11q and 19) correspond to the brightly staining H3K9ac and H3K27ac metaphase bands presented here.

H3K27me3 also shows evidence of regional variation across the genome. An analysis of H3K27me3 across mouse chromosome 17 by ChIP-chip and application of a new algorithm for detecting broad regions of histone modification [41] showed that the modification tends to occur in large regions, designated BLOCs, of average size 43 kb. There are examples of H3K27me3 spreading across large domains in humans, where consistently high levels of H3K27me3 cover the 100- to 200-kb regions encompassing the human *HOX *gene clusters. At a higher level, H3K27me3 BLOCs were found to be more frequent in gene-rich, SINE-rich regions, along with high levels of H3 and H4 acetylation. The authors propose that these regions alternate across the chromosome with gene-poor, SINE-poor, long interspersed nuclear element (LINE)-rich regions with relatively high levels of H3K9me3 and H4K20me3, two histone modifications associated with constitutive heterochromatin [42,43]. As discussed by the authors, this model is not supported by mouse ChIP-seq data [3] analyzed in the same way, or with ENCODE data from human cell lines that showed no evidence for consistent co-localization of H3K27me3 and active histone modifications such as acetylated H3 and H4 and H3K4me3 [44]. The data presented here show that in human metaphase chromosomes, H3K27me3 is preferentially located across defined regions of 10 Mb and above. These regions are not gene-rich, nor does H3K27me3 consistently co-localize with acetylated histones or H3K4me3. However, there is overlap between H3K27me3-rich and H3K4me3/H3K9ac/H3K27ac-rich regions (examples can be seen in Figure 5), showing that, at the highest level, the two chromatin types are not mutually exclusive. As yet we have not been able to align the H3K27me3 banding pattern with any genomic features. H3K27me3 bands do not correspond to the frequency of LINE repeats plotted as 10-Mb windows (results not shown), or to SINE and ALU repeats, which closely correlate, as expected, with gene/CGI density (Additional file 10).

### Functional significance of metaphase chromosome bands

The bands we describe are large, approximately 10 to 50 Mb, and presumably encompass many (perhaps several hundred) smaller chromatin domains, some associated with specific genes and gene clusters and their control elements. A crucial question is whether the bands have any functional significance in their own right, or whether they passively reflect the net level of histone modification among the subdomains that they contain. In assessing this, it is relevant that genes and their control elements make up only a small proportion of the chromatin within a band, with even the most gene-rich band having only approximately 30 genes/Mb. The histone modifications studied here are relatively common and therefore must be mostly located in intergenic chromatin. The difference in gene/CGI density between the most gene-rich and gene-poor domains at 10-Mb resolution is only about 6-fold and differences in repeat density are even less. It is questionable whether differences of this order are sufficient to account for the differences in staining intensity between bands and interbands, with the latter often essentially unstained (that is, comparable to centric heterochromatin). It is also interesting that the banding patterns given by three very different modifications (H3K4me3, H3K9ac and H3K27ac) are so similar. It may be that the banding given by H4K8ac, and other acetylated H4 isoforms, for which the difference in staining intensity between bands and interbands is less extreme, may be a closer reflection of gene/CGI density. It should also be borne in mind that, for some modifications at least, high-level chromosome banding may not be directly determined by DNA sequence elements but by other aspects of chromosome behavior. For example, if interphase chromosome territories are configured so that some regions are accessible to, or share a nuclear location with, subsets of histone modifying enzymes, then one would expect to see large chromosome domains displaying high levels of selected histone modifications, just as we observe.

Our results show that for active modifications (H3K4me3, H3K9ac and H3K27ac) immunofluorescent bands on metaphase chromosomes correspond to enriched regions in the interphase epigenome revealed by ChIP-based approaches [18,40]. The localized persistence of active modifications through mitosis may play a role in determining gene function in the following G1 phase, thereby contributing to the heritability of epigenetic states [45]. This could be done by maintaining a general chromatin property, such as the open chromatin structure found across gene-rich regions of the chromosome [46]. A close analysis of the human transcriptome map (HTM) by Versteeg and colleagues [29] showed that many highly expressed genes are clustered in about 40 gene-rich regions of 10 to 15 Mb in size, designated ridges. Weakly expressed genes tend to cluster in similarly sized, gene poor regions designated antiridges. A quantitative analysis of the properties of ridges and antiridges on chromosomes 1, 6 and 11 in six different human cell lines, all in G1 phase, showed that ridges were consistently less condensed, less spherical and further from the nuclear periphery than antiridges. These properties were not changed by the major differences between cell types in karyotype and gene expression pattern [28]. Ridges often correspond in both position and extent to the metaphase chromosome bands rich in active histone modifications described here (the band at 60 to 70 Mb on chromosome 11p is a good example) [28,29]. It may be that the distribution of histone modifications at metaphase represents part of a mechanism by which the structural properties of gene-rich regions and subregions are maintained through mitosis. Large chromatin regions carrying specific combinations of modified histones could also help establish chromosome territories in the reforming G1 nucleus, perhaps to ensure optimum positioning of gene-rich chromatin [47].

The H3K27me3-rich bands on metaphase chromosomes appear to have no equivalents in interphase, indicating that they are generated by regional adjustment of this modification as cells progress through mitosis. In this context, it is interesting that the intensity of H3K27me3 staining, particularly of the smaller chromosomes such as 19 and 20, is more variable between cells than is that of the other modifications studied here (Additional file 6). Perhaps this is attributable to ongoing demethylation of H3K27 in metaphase cells, with the paler-staining chromosomes being derived from cells that had been blocked in metaphase for longer (up to 4 hours). As noted earlier, close comparison of interphase and metaphase distributions of active modifications suggests that here too there are targeted changes in modification levels, with a selective reduction in interband regions serving to enhance the banding pattern in metaphase. Taken together, these findings suggest that there is widespread remodeling of the epigenome during mitosis. The enzymatic mechanisms responsible for such remodeling can be investigated by selective disruption of candidate enzymes and it will be of particular interest to explore how these mechanisms might be subverted in disease states.

## Conclusions

There is a characteristic distribution of histone modifications across the metaphase genome, giving each chromosome a distinctive immunofluorescent banding pattern. Bands of the active modifications H3K4me3, H3K9ac and H3K27ac are virtually indistinguishable, but differ from bands of H3K27me3. Bands are consistent between cell lines of the same type and, for H3K4me3 at least, between two different cell lineages. There is a close correlation between bands of active modifications and gene/CGI density, SINE density and transcript levels in interphase, though none of these parameters alone provides a complete explanation for the location and relative staining intensities of the different bands. The functional, or DNA sequence properties that correlate with the H3K27me3 bands remain mysterious. For H3K4me3, H3K9ac and H3K27ac, the metaphase banding resembles their distribution at interphase, whereas for H3K27me3 metaphase and interphase distributions are different. The results provide evidence of extensive remodeling of the epigenome as cells enter mitosis, even for modifications where the resemblance between interphase and metaphase distributions is clear.

## Materials and methods

### Cell lines

Immortalized, Epstein Barr virus-transformed LCLs from healthy, karyotypically normal individuals were established in-house and maintained at 37°C, 5% CO_2 _in RPMI 1640 medium, 10% fetal bovine serum, supplemented with L-glutamine (2 mM) and penicillin/streptomycin (all additives from Gibco (Gibco, Grand Island, NY, USA)). These lines retain a normal diploid karyotype over many years in culture and provide a consistent source of experimental material. All results presented here are from two individuals, one male (line AH) and one female (line VM).

### Antisera

Rabbit polyclonal antisera used for immunolabeling are listed in Additional file 8. In-house antisera were prepared by immunization with synthetic peptides as previously described [35,48]. The specificity of all antisera was confirmed by inhibition ELISA using native histones immobilized on microtiter plates and a selection of synthetic peptides, as described previously [48].

### Preparation of chromosome spreads and immunostaining

Cells in exponential growth were treated for 2 hours with colcemid (KaryoMax, Gibco) at 0.1 μg/ml. Cells were pelleted by centrifugation at 1,200 rpm (Chillspin, MSE, London, UK) for 5 minutes at 4°C, washed twice with cold phosphate buffered saline, resuspended in 75 mM KCl at 2 × 10^5 ^cells/ml and left at room temperature for 10 minutes. Aliquots (200 μl) of the swollen cell suspension were spun onto glass slides at 1,800 rpm for 10 minutes in a Shandon Cytospin 4. Slides were then immersed for 10 minutes at room temperature in KCM buffer (120 mM KCl, 20 mM NaCl, 10 mM Tris/HCl pH 8.0, 0.5 mM EDTA, 0.1% Triton X-100). Immunolabeling was carried out for 1 hour at 4°C, as described previously [9], with antisera diluted 200- to 400-fold in KCM supplemented with 1 to 1.5% BSA (Sigma-Aldrich, Dorset, UK). For all labelings, the secondary antibody was fluorescein isothiocyanate (FITC)-conjugated goat anti-rabbit immunoglobulin (Sigma F1262) diluted 150-fold in KCM, 1% BSA. Slides were washed twice in KCM (5 minutes at room temperature), fixed in 4% (v/v) formaldehyde (10 minutes, room temperature), rinsed in deionized water and mounted in Vector Shield (Vector Lab, Peterborough, UK) supplemented with DAPI (Sigma) at 2 μg/ml.

### Chromosome identification and karyotyping

Labeled slides were visualized on a Zeiss Axioplan 2 epifluorescence microscope and potentially suitable metaphases spreads captured using Smart Capture software (Digital Scientific, Cambridge, UK). The filmstrip obtained was screened for the best spreads and poor quality spreads discarded. The coordinates of each spread were recorded using an England Finder(tm) graticule. Each identified spread was recaptured at the West Midlands Regional Genetics Laboratory on a Zeiss microscope and images stored in the ISIS software (Metasystems, Altlussheim, Germany). Karyotyping was based on reverse DAPI staining, which gives a conventional G-banding pattern, using the standard chromosome identification criteria used for clinical diagnostic work. A printout of each karyotype was produced and each chromosome located in the spread and numbered. Immunofluorescent karyotypes were then reconstituted in Smart Type software (Digital Scientific) to reveal the distribution of histone modifications across identified chromosomes.

### Chromosome scanning and data analysis

Twelve representative examples of chromosome 1 (that is, 24 chromatid arms) were scanned for fluorescence intensity using the GraphPolygon Extension of IPLab in SmartCapture. The centromere of each chromosome was manually marked by adding a line of blue pixels, and each chromatid arm was manually tracked from pter, to centromere, to qter. GraphPolygon data comprised color intensities averaged over the pixels on each segment perpendicular to the manually tracked medial axis of the chromatid (for a line of specified width, which is slightly less than the chromatid arm). Numerical pixel values were obtained for green (FITC), red (DAPI) and blue (manually annotated centromere). The relative longitudinal position of each segment of each chromatid was then normalized to the actual length of chromosome 1 (247 Mb). The segment data from each chromatid were first anchored at the centromere (120 Mb), whose position was determined by the maximal level of pixel staining in the blue channel. Color intensity of FITC and DAPI staining was normalized for each chromatid arm as a percentage of the maximum staining for the relevant color. The normalized longitudinal segment positions were then grouped into 25 10-Mb windows and the mean and standard deviation of FITC intensity and DAPI intensity were calculated for each window.

ChIP-seq data from the Bernstein laboratory at the Broad Institute of MIT and Harvard were downloaded from the ENCODE data coordination center at UCSC [13]. Signal data for the lymphoblastoid line GM12878 was averaged for each 10-Mb window for comparison with our data. The CGI distributions presented in this paper are all calculated from the data recently generated and compiled by Illingworth *et al*. [49] using CXXC affinity purification and deep sequencing.

### Microarray expression analysis

RNA was extracted and purified from log-phase lymphoblastoid cells using the RNeasy kit with DNase digestion (Qiagen, Crawley, West Sussex, UK), according to the manufacturer's instructions. cDNA was synthesized using the Superscript double-stranded DNA synthesis kit (Invitrogen, Paisley, UK), cleaned up by RNase A treatment (Invitrogen; 4 μg per 150 μl reaction) followed by phenol:chloroform extraction and ethanol precipitation. Samples were labeled with cy3, hybridized to a 12 × 135 k HD2 expression array (Roche Nimblegen, Madison, Wisconsin, USA); containing 3 probes per gene for 44,049 human genes) and scanned (GenePix 4000B, Molecular Devices, Sunnyvale, CA, USA). Data were extracted, normalized by quantile normalization and gene calls generated by the robust multichip average algorithm using Nimblescan (Roche Nimblegen). Normalized expression data from three biological replicates is available at the Gene Expression Omnibus [50], accession number [GEO:GSE24459]; the data cover the entire human genome and are limited to RefSeq gene sequences. For comparison with H3K4me 3 levels and gene/CGI density, transcription from RefSeq genes was summed within 10-Mb windows across chromosome 1.

## Abbreviations

BSA: bovine serum albumin; CGI: CpG island; ChIP: chromatin immunoprecipitation; DAPI: 4,6-diamino-2-phenyl-indole; ELISA: enzyme-linked immunosorbant assay; FITC: fluorescein isothiocyanate; LCL: lymphoblastoid cell line; LINE: long interspersed nuclear element; SINE: short interspersed nuclear element.

## Competing interests

The authors declare that they have no competing interests.

## Authors' contributions

ET, FM, JAH and BMT conceived and designed experiments; ET, FM, JAH and PP performed experiments; ET, FM, JAH, PP, LPO and BMT analyzed data; RSI, AMRT, VD and LPO contributed reagents, materials and analytical/technical expertise; ET, FM, JAH and BMT wrote the paper. All authors read and approved the final manuscript.

## Supplementary Material

Additional file 1 **Figure showing relative lengths of human chromosomes with and without fixation in methanol acetic acid**. Click here for file

Additional file2 **Figure showing centromeric indices of human chromosomes with and without fixation in methanol acetic acid**. Click here for file

Additional file 3 **Figure showing alignment of individual metaphase chromosomes immunostained for H3K4me3 from five different chromosome spreads**. Click here for file

Additional file 4 **Figure showing alignment of individual metaphase chromosomes immunostained for H3K27ac from six different chromosome spreads**. Click here for file

Additional file 5 **Figure showing alignment of individual metaphase chromosomes immunostained for H3K9ac from two different chromosome spreads**. Click here for file

Additional file 6 **Figure showing alignment of individual metaphase chromosomes immunostained for H3K27me3 from six different chromosome spreads**. Click here for file

Additional file 7 **Figure showing the correspondence between gene density, CpG island density and H3K27me3 levels across human metaphase chromosomes**. Click here for file

Additional file 8 **Table showing antibodies used for labeling and their origins**. Click here for file

Additional file 9 **Immunostained karyotype showing the distribution of H3K4me3 across human fibroblast chromosomes**. Click here for file

Additional file 10 **Table showing correlations between H3K4me3 levels across metaphase chromosome 1, gene-, CGI- and repeat-frequencies and various properties of the interphase epigenome**. Click here for file

## References

[B1] Fan S Zhang X CpG island methylation pattern in different human tissues and its correlation with gene expression. Biochem Biophys Res Commun 2009 383 421 425 10.1016/j.bbrc.2009.04.023 19364493

[B2] Meissner A Mikkelsen TS Gu H Wernig M Hanna J Sivachenko A Zhang X Bernstein BE Nusbaum C Jaffe DB Gnirke A Jaenisch R Lander ES Genome-scale DNA methylation maps of pluripotent and differentiated cells. Nature 2008 454 766 770 18600261 10.1038/nature07107 PMC2896277

[B3] Mikkelsen TS Ku M Jaffe DB Issac B Lieberman E Giannoukos G Alvarez P Brockman W Kim TK Koche RP Lee W Mendenhall E O'Donovan A Presser A Russ C Xie X Meissner A Wernig M Jaenisch R Nusbaum C Lander ES Bernstein BE Genome-wide maps of chromatin state in pluripotent and lineage-committed cells. Nature 2007 448 553 560 10.1038/nature06008 17603471 PMC2921165

[B4] Schones DE Zhao K Genome-wide approaches to studying chromatin modifications. Nat Rev Genet 2008 9 179 191 10.1038/nrg2270 18250624 PMC10882563

[B5] Peters AH Kubicek S Mechtler K O'Sullivan RJ Derijck AA Perez-Burgos L Kohlmaier A Opravil S Tachibana M Shinkai Y Martens JH Jenuwein T Partitioning and plasticity of repressive histone methylation states in mammalian chromatin. Mol Cell 2003 12 1577 1589 10.1016/S1097-2765(03)00477-5 14690609

[B6] Kourmouli N Jeppesen P Mahadevhaiah S Burgoyne P Wu R Gilbert DM Bongiorni S Prantera G Fanti L Pimpinelli S Shi W Fundele R Singh PB Heterochromatin and tri-methylated lysine 20 of histone H4 in animals. J Cell Sci 2004 117 2491 2501 10.1242/jcs.01238 15128874

[B7] Jin W Lamb JC Zhang W Kolano B Birchler JA Jiang J Histone modifications associated with both A and B chromosomes of maize. Chromosome Res 2008 16 1203 1214 10.1007/s10577-008-1269-8 18987983

[B8] Jeppesen P Turner BM The inactive X chromosome in female mammals is distinguished by a lack of histone H4 acetylation, a cytogenetic marker for gene expression. Cell 1993 74 281 289 10.1016/0092-8674(93)90419-Q 8343956

[B9] Keohane AM O'Neill LP Belyaev ND Lavender JS Turner BM X-Inactivation and histone H4 acetylation in embryonic stem cells. Dev Biol 1996 180 618 630 10.1006/dbio.1996.0333 8954732

[B10] Shi J Dawe RK Partitioning of the maize epigenome by the number of methyl groups on histone H3 lysines 9 and 27. Genetics 2006 173 1571 1583 10.1534/genetics.106.056853 16624902 PMC1526679

[B11] Chadwick BP Willard HF Multiple spatially distinct types of facultative heterochromatin on the human inactive X chromosome. Proc Natl Acad Sci USA 2004 101 17450 17455 10.1073/pnas.0408021101 15574503 PMC534659

[B12] Jeppesen P Histone acetylation: a possible mechanism for the inheritance of cell memory at mitosis. Bioessays 1997 19 67 74 10.1002/bies.950190111 9008418

[B13] ENCODE Project at UCSC. http://genome.ucsc.edu/ENCODE/

[B14] Comings DE Avelino E Mechanisms of chromosome banding. II. Evidence that histones are not involved. Exp Cell Res 1974 86 202 206 10.1016/0014-4827(74)90674-0 4133907

[B15] Stenman S Rosenqvist M Ringertz NR Preparation and spread of unfixed metaphase chromosomes for immunofluorescence staining of nuclear antigens. Exp Cell Res 1975 90 87 94 10.1016/0014-4827(75)90360-2 164358

[B16] Jeppesen P Mitchell A Turner B Perry P Antibodies to defined histone epitopes reveal variations in chromatin conformation and underacetylation of centric heterochromatin in human metaphase chromosomes. Chromosoma 1992 101 322 332 10.1007/BF00346011 1374304

[B17] Craig JM Bickmore WA Chromosome bands - flavours to savour. Bioessays 1993 15 349 354 10.1002/bies.950150510 8343145

[B18] Bernstein BE Meissner A Lander ES The mammalian epigenome. Cell 2007 128 669 681 10.1016/j.cell.2007.01.033 17320505

[B19] Ingvarsdottir K Edwards C Lee MG Lee JS Schultz DC Shilatifard A Shiekhattar R Berger SL Histone H3 K4 demethylation during activation and attenuation of GAL1 transcription in *Saccharomyces cerevisiae*. Mol Cell Biol 2007 27 7856 7864 10.1128/MCB.00801-07 17875926 PMC2169161

[B20] Regha K Sloane MA Huang R Pauler FM Warczok KE Melikant B Radolf M Martens JH Schotta G Jenuwein T Barlow DP Active and repressive chromatin are interspersed without spreading in an imprinted gene cluster in the mammalian genome. Mol Cell 2007 27 353 366 10.1016/j.molcel.2007.06.024 17679087 PMC2847180

[B21] Wei G Wei L Zhu J Zang C Hu-Li J Yao Z Cui K Kanno Y Roh TY Watford WT Schones DE Peng W Sun HW Paul WE O'Shea JJ Zhao K Global mapping of H3K4me3 and H3K27me3 reveals specificity and plasticity in lineage fate determination of differentiating CD4+ T cells. Immunity 2009 30 155 167 10.1016/j.immuni.2008.12.009 19144320 PMC2722509

[B22] Tie F Banerjee R Stratton CA Prasad-Sinha J Stepanik V Zlobin A Diaz MO Scacheri PC Harte PJ CBP-mediated acetylation of histone H3 lysine 27 antagonizes *Drosophila *Polycomb silencing. Development 2009 136 3131 3141 10.1242/dev.037127 19700617 PMC2730368

[B23] Wang Z Zang C Rosenfeld JA Schones DE Barski A Cuddapah S Cui K Roh TY Peng W Zhang MQ Zhao K Combinatorial patterns of histone acetylations and methylations in the human genome. Nat Genet 2008 40 897 903 10.1038/ng.154 18552846 PMC2769248

[B24] Margueron R Li G Sarma K Blais A Zavadil J Woodcock CL Dynlacht BD Reinberg D Ezh1 and Ezh2 maintain repressive chromatin through different mechanisms. Mol Cell 2008 32 503 518 10.1016/j.molcel.2008.11.004 19026781 PMC3641558

[B25] Shen X Liu Y Hsu YJ Fujiwara Y Kim J Mao X Yuan GC Orkin SH EZH1 mediates methylation on histone H3 lysine 27 and complements EZH2 in maintaining stem cell identity and executing pluripotency. Mol Cell 2008 32 491 502 10.1016/j.molcel.2008.10.016 19026780 PMC2630502

[B26] Hansen KH Bracken AP Pasini D Dietrich N Gehani SS Monrad A Rappsilber J Lerdrup M Helin K A model for transmission of the H3K27me3 epigenetic mark. Nat Cell Biol 2008 10 1291 1300 10.1038/ncb1787 18931660

[B27] Shen Y Matsuno Y Fouse SD Rao N Root S Xu R Pellegrini M Riggs AD Fan G X-inactivation in female human embryonic stem cells is in a nonrandom pattern and prone to epigenetic alterations. Proc Natl Acad Sci USA 2008 105 4709 4714 10.1073/pnas.0712018105 18339804 PMC2290804

[B28] Goetze S Mateos-Langerak J Gierman HJ de Leeuw W Giromus O Indemans MH Koster J Ondrej V Versteeg R van Driel R The three-dimensional structure of human interphase chromosomes is related to the transcriptome map. Mol Cell Biol 2007 27 4475 4487 10.1128/MCB.00208-07 17420274 PMC1900058

[B29] Versteeg R van Schaik BD van Batenburg MF Roos M Monajemi R Caron H Bussemaker HJ van Kampen AH The human transcriptome map reveals extremes in gene density, intron length, GC content, and repeat pattern for domains of highly and weakly expressed genes. Genome Res 2003 13 1998 2004 10.1101/gr.1649303 12915492 PMC403669

[B30] Gilbert N Gilchrist S Bickmore WA Chromatin organization in the mammalian nucleus. Int Rev Cytol 2005 242 283 336 10.1016/S0074-7696(04)42007-5 15598472

[B31] Bird AP CpG-rich islands and the function of DNA methylation. Nature 1986 321 209 213 10.1038/321209a0 2423876

[B32] Illingworth RS Bird AP CpG islands - 'a rough guide'. FEBS Lett 2009 583 1713 1720 10.1016/j.febslet.2009.04.012 19376112

[B33] ENCODE Project Consortium Birney E Stamatoyannopoulos JA Dutta A Guigó R Gingeras TR Margulies EH Weng Z Snyder M Dermitzakis ET Thurman RE Kuehn MS Taylor CM Neph S Koch CM Asthana S Malhotra A Adzhubei I Greenbaum JA Andrews RM Flicek P Boyle PJ Cao H Carter NP Clelland GK Davis S Day N Dhami P Dillon SC Dorschner MO Identification and analysis of functional elements in 1% of the human genome by the ENCODE pilot project. Nature 2007 447 799 816 10.1038/nature05874 17571346 PMC2212820

[B34] Patzlaff JS Terrenoire E Turner BM Earnshaw WC Paulson JR Acetylation of core histones in response to HDAC inhibitors is diminished in mitotic HeLa cells. Exp Cell Res 2010 316 2123 2135 10.1016/j.yexcr.2010.05.003 20452346 PMC2938188

[B35] Turner BM Fellows G Specific antibodies reveal ordered and cell-cycle-related use of histone-H4 acetylation sites in mammalian cells. Eur J Biochem 1989 179 131 139 10.1111/j.1432-1033.1989.tb14530.x 2917555

[B36] Weber M Hellmann I Stadler MB Ramos L Paabo S Rebhan M Schubeler D Distribution, silencing potential and evolutionary impact of promoter DNA methylation in the human genome. Nat Genet 2007 39 457 466 10.1038/ng1990 17334365

[B37] Ooi SK Qiu C Bernstein E Li K Jia D Yang Z Erdjument-Bromage H Tempst P Lin SP Allis CD Cheng X Bestor TH DNMT3L connects unmethylated lysine 4 of histone H3 to de novo methylation of DNA. Nature 2007 448 714 717 10.1038/nature05987 17687327 PMC2650820

[B38] Turner BM Birley AJ Lavender J Histone H4 isoforms acetylated at specific lysine residues define individual chromosomes and chromatin domains in *Drosophila *polytene nuclei. Cell 1992 69 375 384 10.1016/0092-8674(92)90417-B 1568251

[B39] Turner BM Franchi L Wallace H Islands of acetylated histone H4 in polytene chromosomes and their relationship to chromatin packaging and transcriptional activity. J Cell Sci 1990 96 335 346 2211873 10.1242/jcs.96.2.335

[B40] Roh TY Cuddapah S Zhao K Active chromatin domains are defined by acetylation islands revealed by genome-wide mapping. Genes Dev 2005 19 542 552 10.1101/gad.1272505 15706033 PMC551575

[B41] Pauler FM Sloane MA Huang R Regha K Koerner MV Tamir I Sommer A Aszodi A Jenuwein T Barlow DP H3K27me3 forms BLOCs over silent genes and intergenic regions and specifies a histone banding pattern on a mouse autosomal chromosome. Genome Res 2009 19 221 233 10.1101/gr.080861.108 19047520 PMC2652204

[B42] Schotta G Lachner M Sarma K Ebert A Sengupta R Reuter G Reinberg D Jenuwein T A silencing pathway to induce H3-K9 and H4-K20 trimethylation at constitutive heterochromatin. Genes Dev 2004 18 1251 1262 10.1101/gad.300704 15145825 PMC420351

[B43] Martens JH O'Sullivan RJ Braunschweig U Opravil S Radolf M Steinlein P Jenuwein T The profile of repeat-associated histone lysine methylation states in the mouse epigenome. EMBO J 2005 24 800 812 10.1038/sj.emboj.7600545 15678104 PMC549616

[B44] Thurman RE Day N Noble WS Stamatoyannopoulos JA Identification of higher-order functional domains in the human ENCODE regions. Genome Res 2007 17 917 927 10.1101/gr.6081407 17568007 PMC1891350

[B45] Probst AV Dunleavy E Almouzni G Epigenetic inheritance during the cell cycle. Nat Rev Mol Cell Biol 2009 10 192 206 10.1038/nrm2640 19234478

[B46] Gilbert N Boyle S Fiegler H Woodfine K Carter NP Bickmore WA Chromatin architecture of the human genome: gene-rich domains are enriched in open chromatin fibers. Cell 2004 118 555 566 10.1016/j.cell.2004.08.011 15339661

[B47] Bolzer A Kreth G Solovei I Koehler D Saracoglu K Fauth C Müller S Eils R Cremer C Speicher MR Cremer T Three-dimensional maps of all chromosomes in human male fibroblast nuclei and prometaphase rosettes. PLoS Biol 2005 3 e157 10.1371/journal.pbio.0030157 15839726 PMC1084335

[B48] White DA Belyaev ND Turner BM Preparation of site-specific antibodies to acetylated histones. Methods 1999 19 417 424 10.1006/meth.1999.0878 10579937

[B49] Illingworth RS Gruenewald-Schneider U Webb S Kerr ARW James KD Turner DJ Smith C Harrison DJ Andrews R Bird AP Orphan CpG islands identify numerous conserved promoters in the mammalian genome. PLoS Genet 2010 6 e1001134 10.1371/journal.pgen.1001134 20885785 PMC2944787

[B50] Gene Expression Omnibus (GEO) genomics data repository. http://www.ncbi.nlm.nih.gov/geo/

[B51] O'Neill LP Spotswood HT Fernando M Turner BM Differential loss of histone H3 isoforms mono-, di- and tri-methylated at lysine 4 during X-inactivation in female embryonic stem cells. Biol Chem 2008 389 365 370 10.1515/BC.2008.046 18225985

[B52] Perez-Burgos L Peters AH Opravil S Kauer M Mechtler K Jenuwein T Generation and characterization of methyl-lysine histone antibodies. Methods Enzymol 2004 376 234 254 full_text 14975310 10.1016/S0076-6879(03)76016-9

