# Supplementary figures and images for: Immunostaining of modified histones defines high-level features of the human metaphase epigenome
Source: Genome Biol. 2010 Nov 15;11(11):R110. doi: 10.1186/gb-2010-11-11-r110 (PMC3156949; doi:10.1186/gb-2010-11-11-r110)

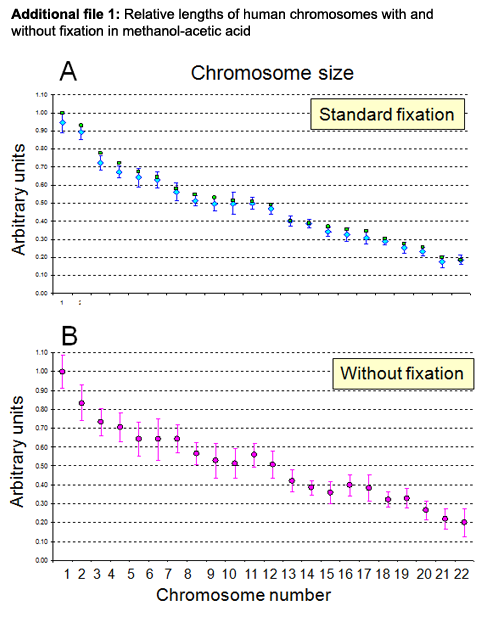

Supplement: Additional file 1 — Figure showing relative lengths of human chromosomes with and without fixation in methanol acetic acid. [file gb-2010-11-11-r110-S1.tiff]

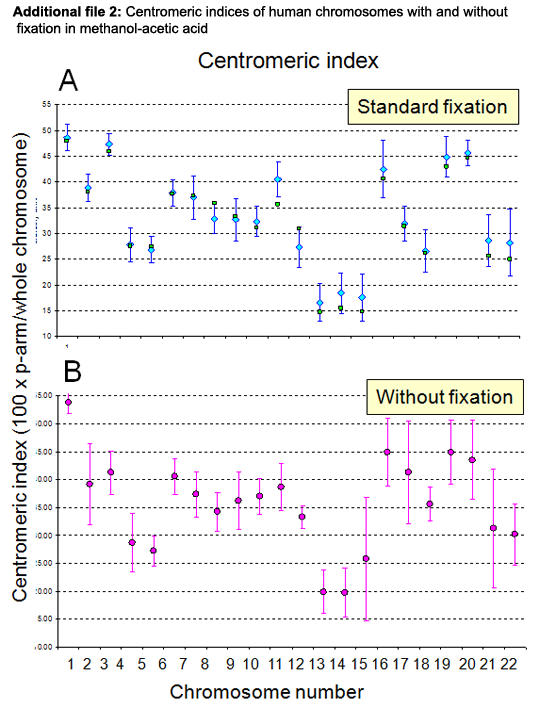

Supplement: Additional file2 — Figure showing centromeric indices of human chromosomes with and without fixation in methanol acetic acid. [file gb-2010-11-11-r110-S2.tiff]

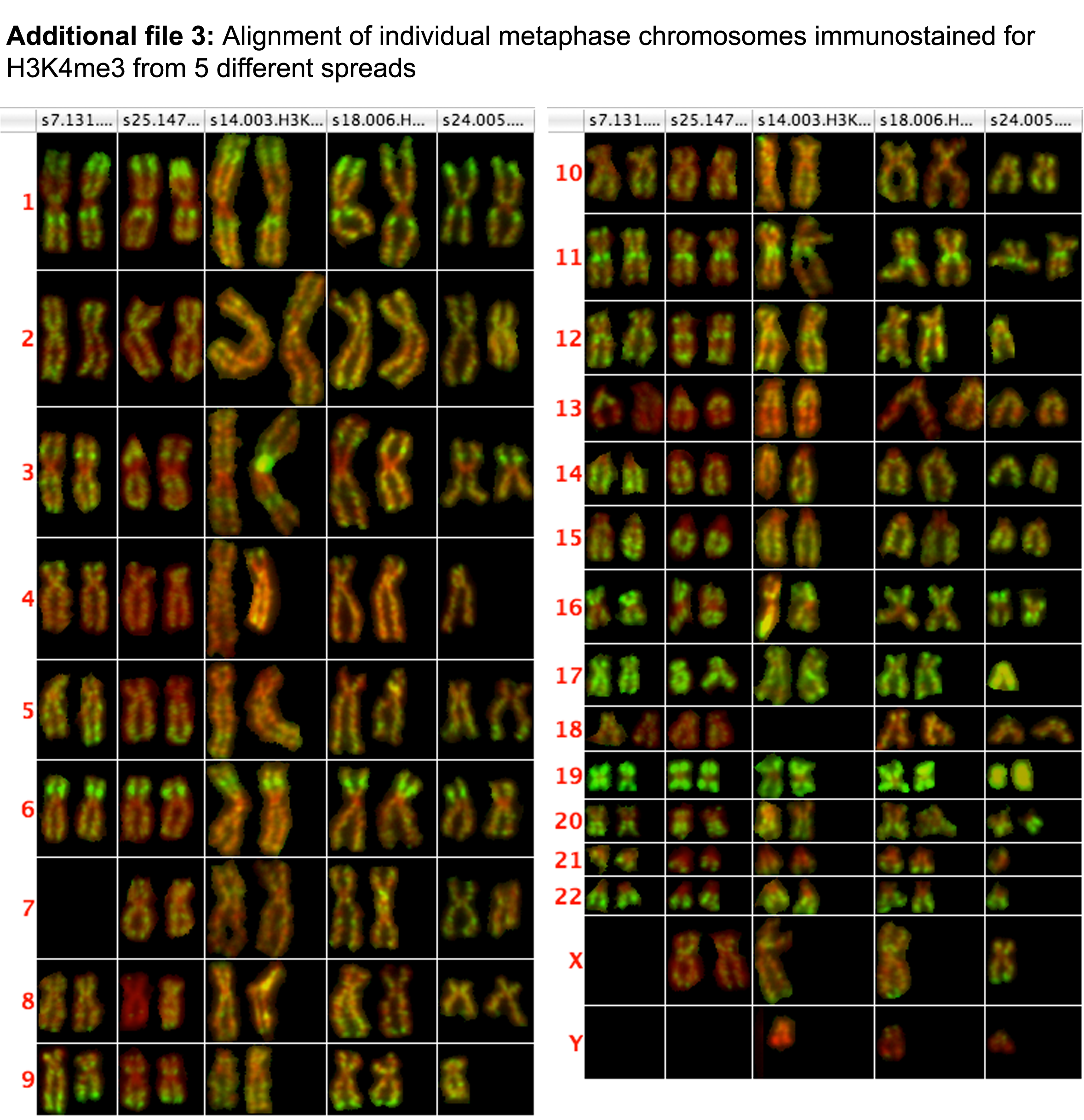

Supplement: Additional file 3 — Figure showing alignment of individual metaphase chromosomes immunostained for H3K4me3 from five different chromosome spreads. [file gb-2010-11-11-r110-S3.tiff]

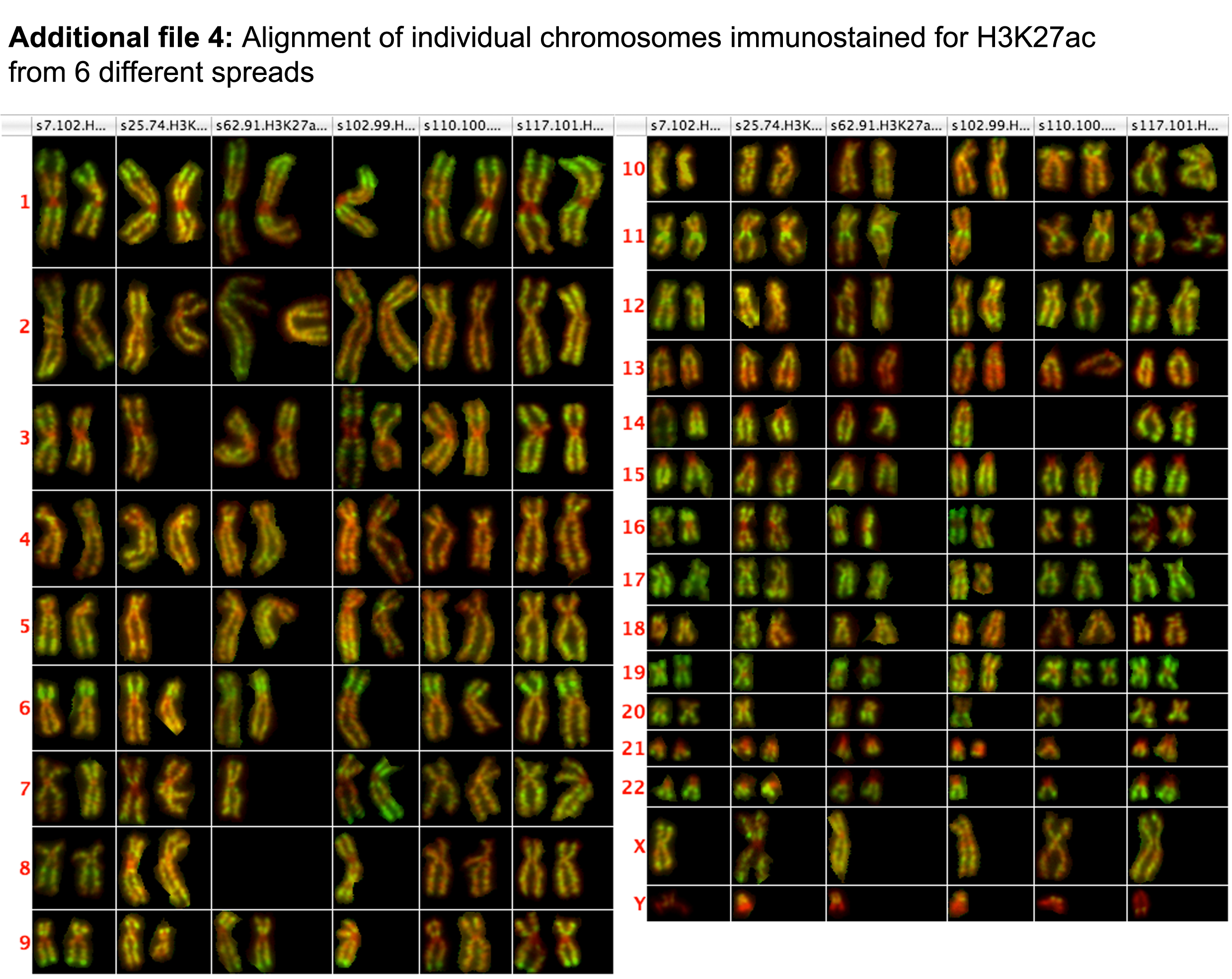

Supplement: Additional file 4 — Figure showing alignment of individual metaphase chromosomes immunostained for H3K27ac from six different chromosome spreads. [file gb-2010-11-11-r110-S4.tiff]

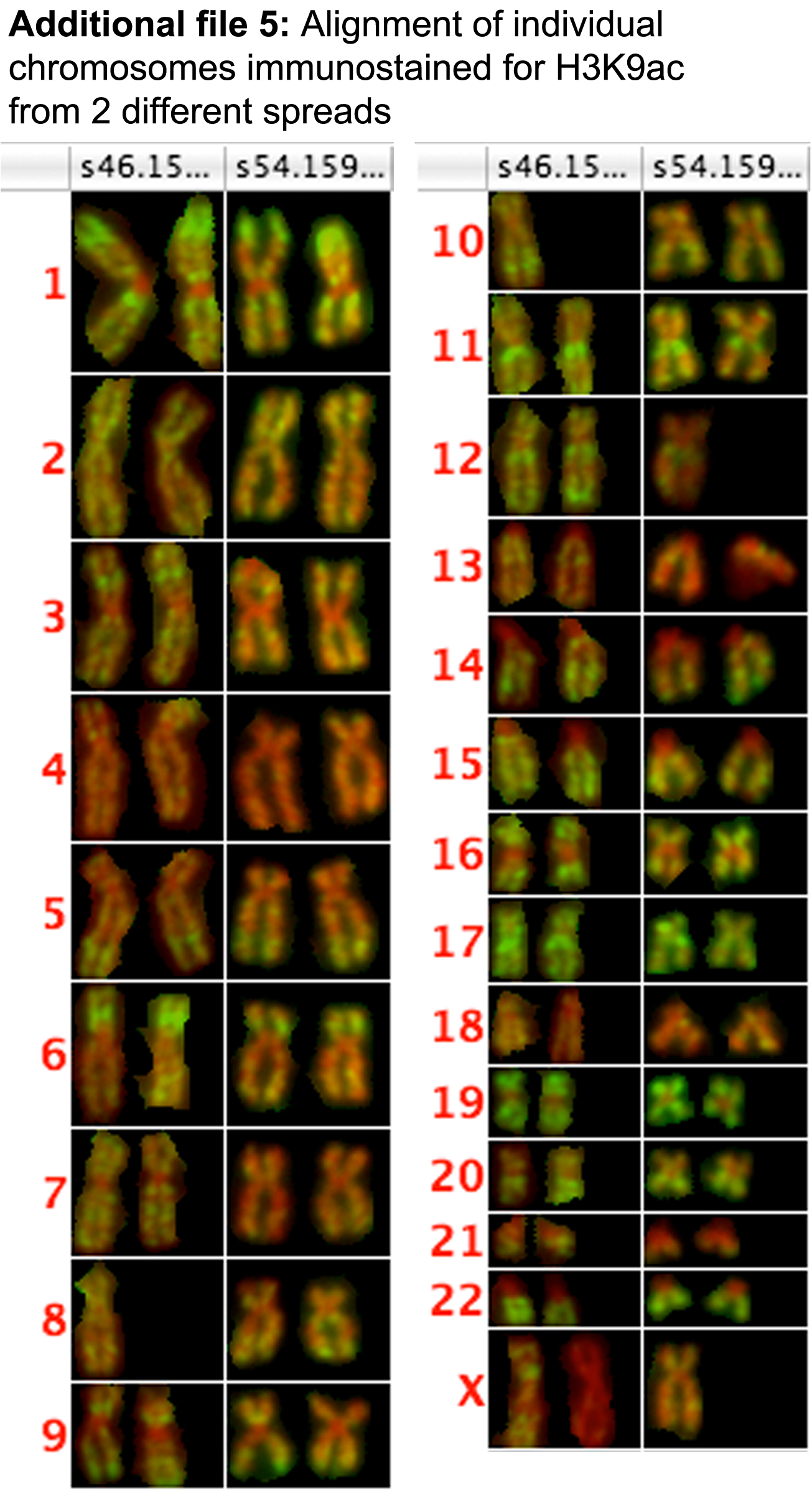

Supplement: Additional file 5 — Figure showing alignment of individual metaphase chromosomes immunostained for H3K9ac from two different chromosome spreads. [file gb-2010-11-11-r110-S5.tiff]

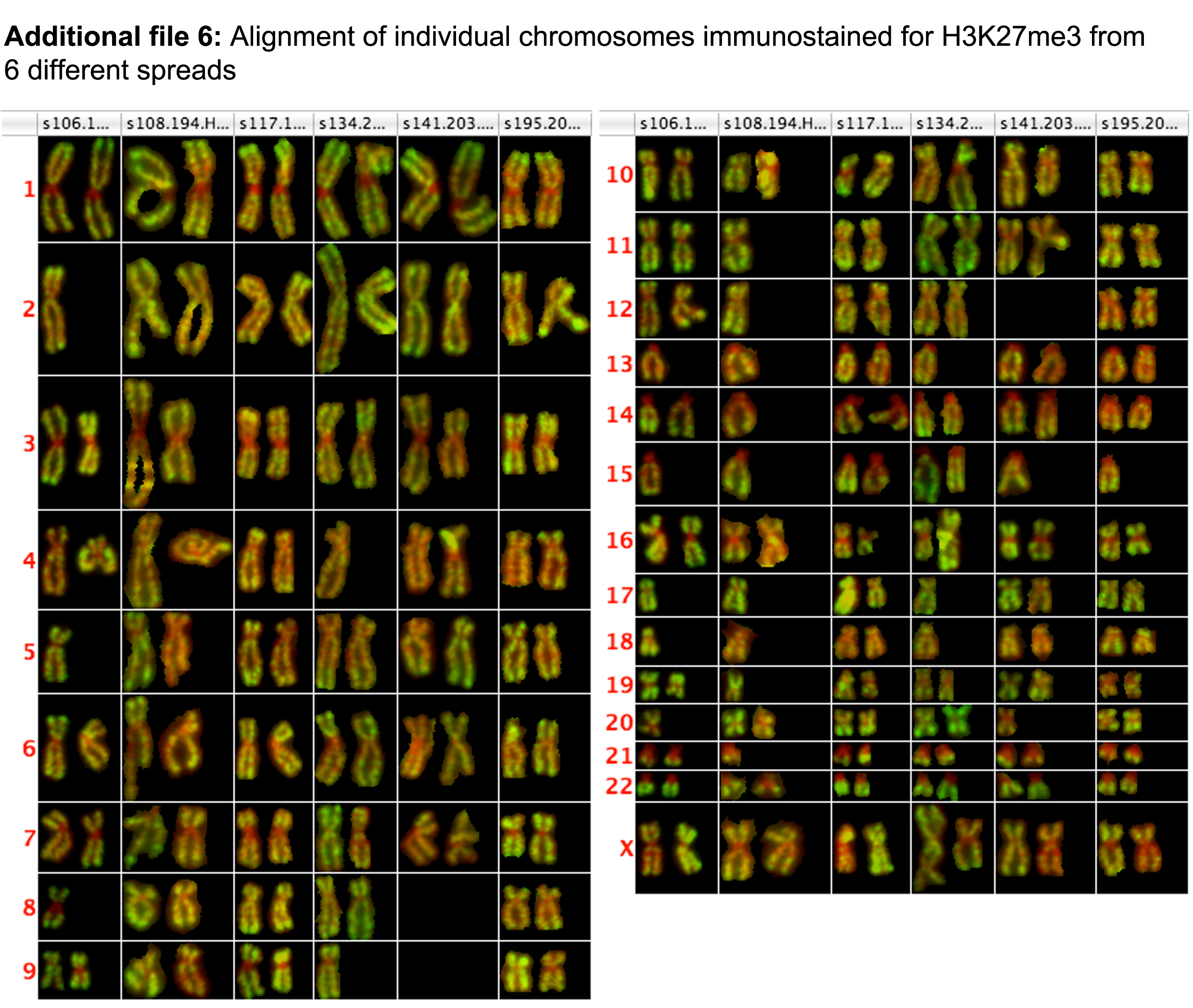

Supplement: Additional file 6 — Figure showing alignment of individual metaphase chromosomes immunostained for H3K27me3 from six different chromosome spreads. [file gb-2010-11-11-r110-S6.tiff]

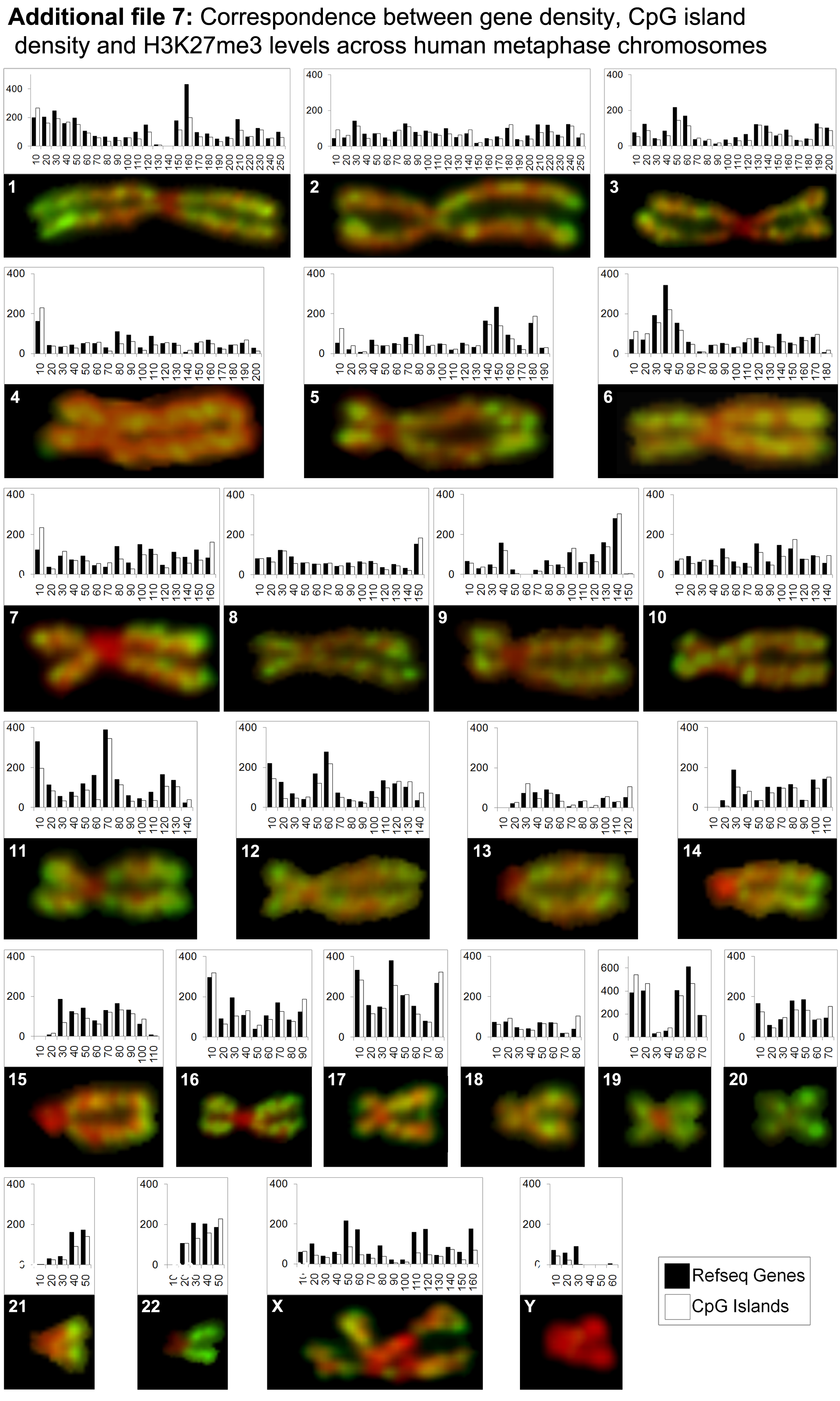

Supplement: Additional file 7 — Figure showing the correspondence between gene density, CpG island density and H3K27me3 levels across human metaphase chromosomes. [file gb-2010-11-11-r110-S7.tiff]

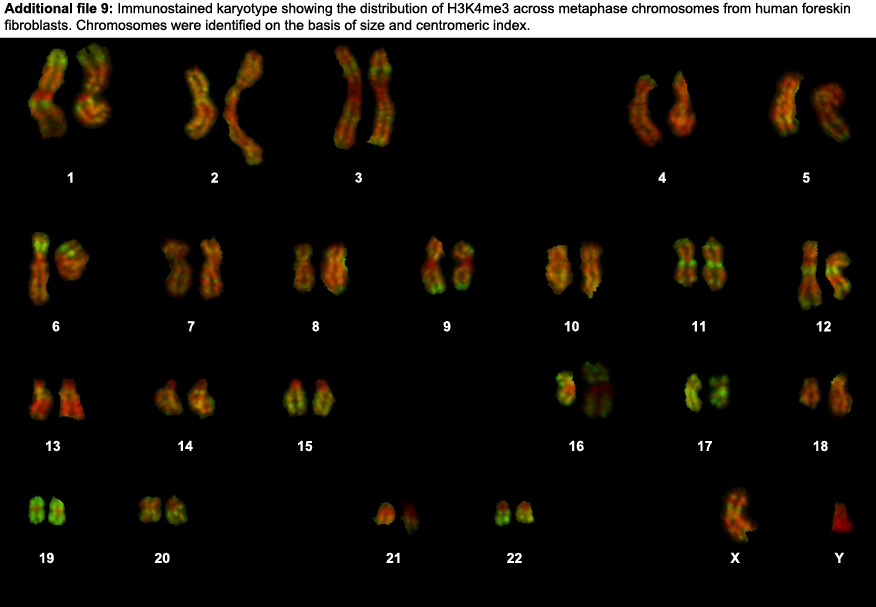

Supplement: Additional file 9 — Immunostained karyotype showing the distribution of H3K4me3 across human fibroblast chromosomes. [file gb-2010-11-11-r110-S9.tiff]
