# Supplementary material for: Immunostaining of modified histones defines high-level features of the human metaphase epigenome
Source: Genome Biol. 2010 Nov 15;11(11):R110. doi: 10.1186/gb-2010-11-11-r110 (PMC3156949; doi:10.1186/gb-2010-11-11-r110)
Supplement: Additional file 8 — Table showing antibodies used for labeling and their origins. [file gb-2010-11-11-r110-S8.doc]

**Additional file 8**

*Terrenoire et al*

ANTIBODIES

| Specificity | Source | Product code | Notes |
| --- | --- | --- | --- |
| H3K4me3 | *In house* | R183 | O’Neill et al. 2008 [51] |
| H3K9ac | Abcam | Ab10812 |  |
| H3K27ac | Abcam | Ab4729 |  |
| H3K27me3 | T. Jenuwein lab,  IMP Vienna |  | Perez-Burgos et al. 2004 [52] |
| H3K27me3 | Millipore | 07-449 |  |
| H4K8ac | *In house* | R403 |  |
| H4K20me3 | Abcam | Ab9053-100 |  |
