# Supplementary material for: Immunostaining of modified histones defines high-level features of the human metaphase epigenome
Source: Genome Biol. 2010 Nov 15;11(11):R110. doi: 10.1186/gb-2010-11-11-r110 (PMC3156949; doi:10.1186/gb-2010-11-11-r110)
Supplement: Additional file 10 — Table showing correlations between H3K4me3 levels across metaphase chromosome 1, gene-, CGI- and repeat-frequencies and various properties of the interphase epigenome. [file gb-2010-11-11-r110-S10.docx]

**Additional file 10**

*Terrenoire et al*

*Pairwise correlations between levels of H3K4me3 on human chromosome 1 at metaphase and various DNA sequence elements, transcription and histone modifications at interphase.*

|  | **Pearson correlation (r value)** | | | | | | | | | |
| --- | --- | --- | --- | --- | --- | --- | --- | --- | --- | --- |
|  | M phase H3K4me3 | Genes | CGI | Transc’n | ENCODE H3K4me3 | ENCODE H3K27ac | ENCODE H3K27me3 | ALU | SINE | LINE |
| M phase H3K4me3 | 1.00 | - | - | - | - | - | - | - | - | - |
| Genes | 0.70 | 1.00 | - | - | - | - | - | - | - | - |
| CGI | 0.68 | 0.83 | 1.00 | - | - | - | - | - | - | - |
| Transcription | 0.73 | 0.92 | 0.87 | 1.00 | - | - | - | - | - | - |
| ENC H3K4me3 | 0.74 | 0.87 | 0.79 | 0.93 | 1.00 | - | - | - | - | - |
| ENC H3K27ac | 0.78 | 0.91 | 0.83 | 0.87 | 0.94 | 1.00 | - | - | - | - |
| ENC H3K27me3 | 0.56 | 0.60 | 0.56 | 0.56 | 0.80 | 0.71 | 1.00 | - | - | - |
| ALU | 0.80 | 0.73 | 0.85 | 0.76 | 0.79 | 0.86 | 0.67 | 1.00 | - | - |
| SINE | 0.74 | 0.65 | 0.76 | 0.65 | 0.73 | 0.80 | 0.70 | 0.96 | 1.00 | - |
| LINE | 0.36 | 0.29 | 0.27 | 0.11 | 0.44 | 0.45 | 0.69 | 0.48 | 0.59 | 1.00 |

The correlations (Pearson r values) between pairs of variables are shown. Chromosome 1 was divided into 25 x10Mb windows to cover the whole chromosome. The sum of each variable was calculated within each window. Each r value represents the correlation between a pair of variables across all 25 windows. Values for gene and CGI content are shown in Fig.3, total transcription and H3K4me3 levels at metaphase (from chromosome scanning) in Fig.4 and ENCODE levels of H3K4me3, H3K27ac and H3K27me3 at interphase are shown in Fig.5. Repeat masker-defined repeats were taken from UCSC (hg18) human genome build (<http://genome.ucsc.edu>) and allocated to the same10Mb windows as the other parameters for correlation analysis.
